# The bilaterian roots of cordon-bleu

**DOI:** 10.1186/1756-0500-6-393

**Published:** 2013-09-30

**Authors:** Jörg Schultz, Niklas Terhoeven

**Affiliations:** 1 Department of Bioinformatics, Biocenter, University Würzburg, Am Hubland, Würzburg 97074, Germany

**Keywords:** Actin nucleation, WH2 domain, Cobl domain, Gene duplication

## Abstract

**Background:**

The actin cytoskeleton is essential for many physiological processes of eukaryotic cells. The emergence of new actin fibers is initiated by actin nucleators. Whereas most of them are evolutionary old, the cordon-bleu actin nucleator is classified as vertebrate specific.

**Findings:**

Using sensitive methods for sequence similarity detection, we identified homologs of cordon-bleu not only in non-vertebrate chordates but also in arthropods, molluscs, annelids and platyhelminthes. These genes contain only a single WH2 domain and therefore resemble more the vertebrate cordon-bleu related 1 protein than the three WH2 domain containing cordon-bleu. Furthermore, we identified a homolog of the N-terminal, ubiquitin like, cobl domain of cordon-bleu in the cnidarian *Nematostella vectensis*.

**Conclusion:**

Our results suggest that the ur-form of the cordon-bleu protein family evolved already with the emergence of the bilateria by the combination of existing cobl and WH2 domains. Following a vertebrate specific gene-duplication, one copy gained two additional WH2 domains leading to the actin nucleating cordon-bleu. The function of the ur-form of the cordon-bleu protein family is so far unknown. The identification of a homolog in the model organism *Drosophila melanogaster* could facilitate its experimental characterization.

## Findings

The actin cytoskeleton is one of the hallmarks of eukaryotic cells. It is involved in processes like movement, phagocytosis and morphogenesis, to name just a few [1]. Although its roots can be traced back to prokaryotes and archaea [2], eukaryotes have evolved an arsenal of actin associated proteins to orchestrate its functions. One of the key processes to be regulated is the transformation of the globular (G-Actin) to the filamentous form (F-actin). Although this process can happen spontaneously, it is kinetically unfavorable and therefore inefficient. In the eukaryotic cell, this process is supported by different proteins which assist in the nucleation, extension and branching of actin filaments [3,4]. The first described and arguably most prominent is the ARP2/3 complex, which can not only initiate the nucleation of new actin filaments but also their branching. Its origin can be traced back to the last common ancestor of the eukaryotes (LECA) [5]. Within the last years further proteins crucial for the efficient nucleation of actin filaments were identified. The actin nucleating FH2 domain is the hallmark of formins, which can be found throughout the eukaryotic kingdom [6,7]. Only later an additional actin nucleation domain, namely WH2, was characterized [8]. Proteins containing this domain have been identified throughout the eukaryotes and a prokaryotic origin has been suggested [9]. One of the WH2 domain type actin nucleators is cordon-bleu, a protein involved in the development of the central nervous system [10], the neural tube [11] and motile cilia [12]. Contrasting the other so far known actin nucleators, this gene was described as vertebrate specific [11]. This might be unexpected, considering that these evolutionary young proteins are needed for the regulation of a process already present in LECA. We therefore set out to unravel the evolutionary roots of this gene.

WH2 domains are short and divergent and thus hard to identify with standard sequence analysis approaches. We therefore in the first step focused on a second conserved domain in the N-terminus of cordon-bleu, the cobl domain. This domain belongs to the ubiquitin fold and its structure has been solved (PDB 2DAJ). A PSI-Blast search [13] with this sequence identified in the first search a significantly similar hit in *Branchiostoma floridae* (Cephalochordata, GI:260809417, E=2×10^-7^). In the first iteration, hits were found in *Saccoglossus kowalevskii* (Hemichordata, GI:291232830, E=10^-8^) and the sea urchin *Strongylocentrotus purpuratus* (Echinodermata, GI:390346270, E=4×10^-6^). Surprisingly, also proteins of arthropods, e.g. the honey bee *Apis mellifera* (GI:328778502, E=3×10^-5^), and of Lophotrochozoa, namely the oyster *Crassostrea gigas* (GI:405954164, E=0.002), the sea hare *Aplysia californica* (GI:524908855, E=2×10^-7^) and the annelid *Capitella teleta* (GI:443710193, E=8×10^-12^), were hit. The species most distantly related to vertebrates identified in this iteration was *Capsaspora owczarzaki*, belonging to the Ichtyosporea (GI:470321394, E=0.005). In the next iteration, the profile showed significant similarity to further arthropod proteins including *Drosophila melanogaster* "proximal to raf" (GI:17864372, E=10^-7^). Finally, first bona fide ubiquitin like proteins (GI:227343644, *Trypanosoma brucei* E=0.003) were hit. Back searches with e.g. the *Capitella teleta* sequence identified additionally a protein in the platyhelminth *Clonorchis sinensis* (GI: 358341913, E=6×10^-4^). We thus conclude that the cobl domain evolved at least with the emergence of the bilateria.

To identify possible non-bilaterian members of the domain, we performed Hidden Markov Model searches [14] with an 85% non-redundant alignment of the hits identified with PSI-Blast against the proteomes of a placozoa (*Trichoplax adhaerens*[15]), a sponge (*Amphimedon queenslandica*[16]), cnidaria (*Hydra magnipapillata*[17] and *Nematostella vectensis*[18]) and Ctenophora (*Mnemiopsis leidyi*[19]). Indeed, a significantly similar protein was identified in the proteome of *Nematostella vectensis* (GI:156394513, E=3.8×10^-6^). Still, this single occurrence leaves the presence of a cobl domain in the non-metazoan eukaryote *Capsaspora owczarzaki* enigmatic.

But, do these proteins indeed belong to the cordon-bleu protein family? In vertebrates, a paralog of cordon-bleu, cordon-bleu related 1, exists. Whereas the first contains three C-terminal WH2 domains, the latter contains only one. We therefore set out to identify WH2 domains in the cobl domain containing proteins using sensitive HMM to HMM alignments [20,21]. Indeed, a HMM based on the arthropod sequences identified in the first PSI-Blast iteration was significantly similar to the WH2 HMM from Pfam [22] (PF02205; E=4.3×10^-6^). Therefore, these arthropod sequences contain not only the cobl, but also an additional C-terminal WH2 domain. With the same approach, we were able to identify a single WH2 domain at the C-terminus of the oyster protein (E=0.00025). Next, we aligned a HMM based on vertebrate cordon-bleu proteins against one based on the putative Drosophila homologs. In addition to the cobl domain, a significantly (E=0.065) similar region was identified between the C-terminus of both alignments. Taking the human cordon-bleu as reference, this covered positions 1234 to 1247 and thereby at least parts of the C-terminal WH2 domain. This indicates a single WH2 domain in the C-terminus of the Drosophila "proximal to raf" proteins. Despite the sensitivity of these approaches, no WH2 domain was predicted in the *Nematostella vectensis* protein containing a cobl domain. Contrasting, InterPro identified a WH1 (also found in Wiskott-Aldrich syndrome proteins) domain in position 75–186. The cobl domain containing *Capsaspora owczarzaki* protein also lacks a WH2 domain, but contains a WW, a PTB and a CRIB domain. Most interestingly, the latter is also found in WASp proteins.

Taken together, we have shown that the evolutionary roots of the cordon-bleu (related) protein family lie before the first emergence of vertebrates. Its absence in some major metazoan lineages like the Nematoda can be explained by lineage specific losses. Thus, our results suggest the following evolutionary history of cordon-bleu proteins. First, the N-terminal cobl domain evolved from an existing ubiquitin fold. With the emergence of the bilateria, this domain was combined with a single WH2 domain. Finally, with the emergence of the vertebrates, a gene duplication evolved the cordon-bleu related 1 and the cordon-bleu genes. In the latter, two additional WH2 domains were acquired. We were not able to predict whether these new domains arose via an internal duplication or were acquired from another protein as the sequences were too short to calculate a reliable phylogeny.

To perform as actin nucleator, a protein has to bring different monomeric actin molecules in close proximity. In cordon-bleu, this is achieved by the binding of actin to each of the three WH2 domains. Contrasting, cordon-bleu related 1 as well as the ur-form pre-dating the duplication contain only a single WH2 domain. Therefore, it is unlikely that these molecules have actin nucleation capabilities. Thus, this function evolved following the gene duplication. The identification of a candidate ortholog in the model organism *Drosophila melanogaster* could enable the functional characterization of the ur-form of the cordon-bleu protein family. Following, the adaptations leading to the vertebrate specific function of cordon-bleu could be traced. Thus, the cordon-bleu protein family could become a test case to study functional changes following gene duplications.

## Competing interests

The authors declared that they have no competing interests.

## Authors’ contributions

JS designed the study, performed analyses and wrote the Manuscript. NT performed analyses. Both authors read and approved the final manuscript.
